# Influence of Joint Angle on Residual Force Enhancement in Human Plantar Flexors

**DOI:** 10.3389/fphys.2017.00234

**Published:** 2017-04-24

**Authors:** Atsuki Fukutani, Jun Misaki, Tadao Isaka

**Affiliations:** ^1^Faculty of Kinesiology, University of CalgaryCalgary, AB, Canada; ^2^Japan Society for the Promotion of Science, Postdoctoral Fellowships for Research AbroadTokyo, Japan; ^3^Research Organization of Science and Technology, Ritsumeikan UniversityShiga, Japan; ^4^Graduate School of Sport and Health Science, Ritsumeikan UniversityShiga, Japan; ^5^Faculty of Sport and Health Science, Ritsumeikan UniversityShiga, Japan

**Keywords:** plantar flexion, electrical stimulation, fascicle length, pennation angle, muscle length

## Abstract

Compared to pure isometric contractions, isometric muscle force at a given length is larger when the eccentric contraction is conducted before the isometric contraction. This phenomenon is widely known as residual force enhancement, and has been confirmed consistently in isolated muscle experiments. The purpose of this study was to confirm whether residual force enhancement also occurs in human plantar flexors and to examine its joint angle dependence. Eleven men participated in this study. Isometric joint torque was measured in a Control trial (pure isometric contraction) and Residual force enhancement (RFE) trial (isometric contraction after eccentric contraction) at plantar flexion 0° (Short condition) and dorsiflexion 15° (Long condition). Fascicle length and pennation angle of the medial gastrocnemius were measured simultaneously to evaluate the influence of architectural parameters on isometric joint torque. Isometric joint torque observed in the Short condition was not significantly different between the Control and RFE trials (Control: 42.9 ± 8.0 Nm, RFE: 45.1 ± 8.4 Nm) (*p* = 0.200). In contrast, significant differences in isometric joint torque were observed in the Long condition between Control and RFE trials (Control: 40.5 ± 9.3 Nm, RFE: 47.1 ± 10.5 Nm) (*p* = 0.001). Fascicle length and pennation angle were not different between Control and RFE trials in the Short and Long conditions. Isometric joint torque was larger when eccentric contraction was conducted before isometric contraction while architectural differences were not observed, indicating that residual force enhancement occurs in human plantar flexors. However, the influence of residual force enhancement may be limited in dorsiflexed positions because the magnitude of residual force enhancement is considered to be prominent in the descending limb (long muscle length condition) and small in the ascending limb (short muscle length condition) where human plantar flexors operate in plantar flexed positions.

## Introduction

According to the basic characteristics of muscles, force-length relationship (Edman, 1966; Gordon et al., 1966), isometric muscle force at a given muscle length should be consistent. However, isometric muscle force at a given muscle length is higher after active lengthening (i.e., eccentric contraction) than that at the corresponding muscle length during pure isometric contraction (Abbott and Aubert, 1952; Edman et al., 1978). This phenomenon, which cannot be explained by classic cross-bridge theory (Huxley, 1957), is known as residual force enhancement (Herzog et al., 2006; Edman, 2010). Residual force enhancement is a consistently observed phenomenon in sarcomeres (Leonard et al., 2010; Rassier and Pavlov, 2012), myofibrils (Joumaa et al., 2008), single muscle fibers (Edman et al., 1978, 1982), and isolated whole muscles (Schachar et al., 2002; Hisey et al., 2009). From the results of previous studies, magnitude of force enhancement appears to be greater in the descending limb of a force-length relationship (Morgan et al., 2000), dependent on the magnitude of active lengthening (Bullimore et al., 2007), and independent of the velocity of active lengthening (Edman et al., 1978; Lee and Herzog, 2002).

As mentioned above, although many studies have investigated residual force enhancement, studies adopting human experiments have been limited. Recently, Seiberl et al. (2013) and Paternoster et al. (2016) examined the influence of residual force enhancement on human voluntary multi joint movement, and reported partly conflicting results; the former found statistically-significant residual force enhancement while the latter failed to find statistically-significant residual force enhancement, in other words, responders and non-responders existed. In addition, Lee and Herzog (2002) examined the magnitude of residual force enhancement in human adductor pollicis in both voluntary and electrically-evoked contractions, and reported that significant residual force enhancement was confirmed in both voluntary and electrically-evoked contractions. However, in the voluntary contraction condition, the magnitude of residual force enhancement was significantly smaller in a larger active stretching trial, which contradicts one of the major characteristics of residual force enhancement, i.e., that the magnitude of residual force enhancement increases with increasing the magnitude of active stretch (Bullimore et al., 2007). These conflicting results may be caused by relatively unstable joint torque production due to fluctuations in voluntary neural excitation. Therefore, it may be appropriate to study this phenomenon in artificially-evoked contractions instead of in voluntary contractions.

In addition, it should be noted that human muscles, such as knee extensors and plantar flexors, are known to operate at different regions of the force-length relationship (i.e., ascending limb, plateau, and descending limb) within the physiological range of motion (Herzog et al., 1991; Kawakami et al., 1998; Maganaris, 2001). Considering the fact that the magnitude of residual force enhancement is smaller in the ascending limb and larger in descending limb (Morgan et al., 2000), each muscle may show different responses with respect to active stretching. Thus, the responses of each muscle need to be assessed separately. Some previous studies examined whether residual force enhancement occurs in human plantar flexors (Pinniger and Cresswell, 2007; Hahn et al., 2012). However, the authors tested only one joint angle (dorsiflexion (DF) 15° in the case of Pinniger and Cresswell (2007), and DF20° in the case of Hahn et al. (2012)). Because the magnitude of residual force enhancement differs in different muscle length conditions (Morgan et al., 2000), it is unclear whether residual force enhancement occurs in the short muscle length position, i.e., more plantar-flexed position.

With the above in mind, the purpose of this study was to examine whether residual force enhancement occurs in human plantar flexors in both short and long muscle length conditions using electrically-evoked contractions. We defined plantar flexion (PF) 0° as the Short condition and DF15° as the Long condition. We hypothesized that the magnitude of residual force enhancement is smaller or negligible in the Short condition and larger in the Long condition. In addition, architectural parameters (fascicle length and pennation angle of the medial gastrocnemius) were measured simultaneously to confirm whether joint torque differences, i.e., magnitude of residual force enhancement is partly caused by muscle architectural differences because larger joint torque after active stretch may be caused by fascicle length difference and/or smaller pennation angle.

## Materials and methods

### Subjects

Eleven healthy young men (mean ± standard deviation, age: 24.8 ± 3.1 years; height: 1.72 ± 0.04 m; body mass: 65.7 ± 6.3 kg) voluntarily participated in the present study. The purpose and associated risks of the study were explained to each volunteer, and written informed consents were obtained from all participants. The Ethics Committee on Human Research of Ritsumeikan University approved this study (IRB-2016-007), and the study was conducted according to the principles of the Declaration of Helsinki.

### Experimental setup

**Figure 1:**
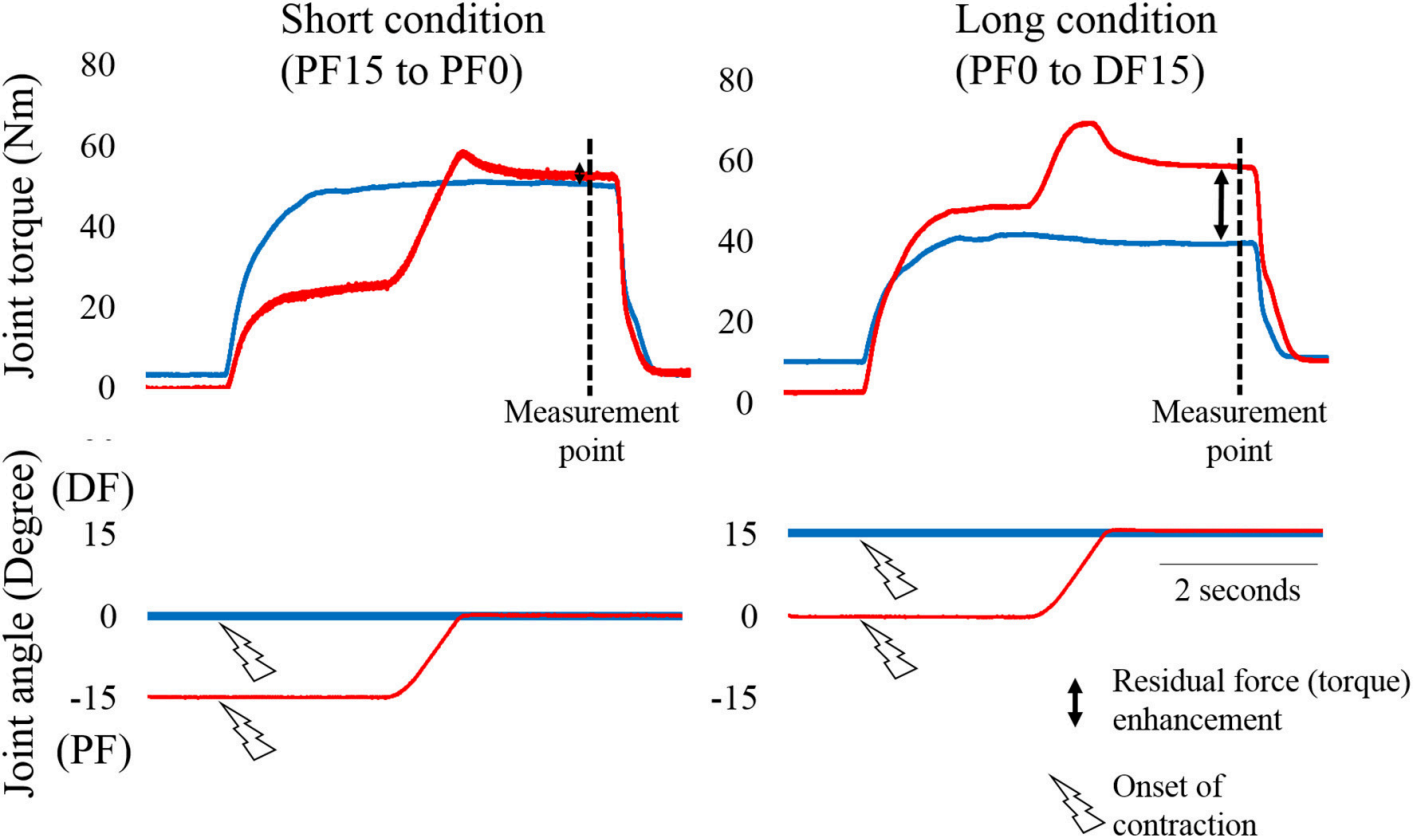
**Typical example of changes in joint torque and joint angle as a function of time**. The left panel shows the Short condition (from plantar flexion (PF) 15° to PF0°) and the right panel shows the Long condition [from PF0° to dorsiflexion (DF) 15°]. The blue line shows the Control trial (pure isometric contraction) and the red line shows the Residual force enhancement trial (isometric contraction after eccentric contraction). Joint toque, fascicle length and pennation angle were measured at the measurement point (i.e., 4.9 s after the onset of contraction). −15 indicates PF15.

Ankle plantar flexors were adopted as the target muscles of this study. Subjects lay in the supine position on a dynamometer (Biodex; SAKAImed, Tokyo, Japan). Ankle, knee, and hip joint angles were set at 0° (i.e., anatomical position). Throughout the experiment, knee and hip joint angles were kept at same joint angle. Ankle joint was fixed on an attachment of the dynamometer. In this study, following two conditions were adopted. The first was the Short condition and the second was the Long condition. In each condition, a residual force enhancement (RFE) trial (i.e., isometric contraction following eccentric contraction) and a Control trial (i.e., purely isometric contraction) were performed to calculate the magnitude of residual force enhancement (i.e., comparing the isometric joint torque obtained at the same joint angle, Figure 1). In this experiment, muscle contractions were evoked using electrical stimulation (SEN-3401; Nihon Kohden, Tokyo, Japan). The stimulation electrodes (4 × 5 cm) were placed on the muscle bellies of the proximal part of the gastrocnemii and the lower side of the soleus. The stimulation parameters were as follows; pulse frequency, 50 Hz; pulse duration, 0.5 ms; train duration, 5 s. To determine the intensity of electrical stimulation, maximal voluntary isometric contraction in plantar flexors was performed with the ankle joint angle at 0°. The highest isometric joint torque recorded during this contraction was set as 100% intensity. The intensity of electrical stimulation was adjusted to evoke 25% of the maximal intensity at the corresponding joint angle. This electrical stimulation intensity was applied to the all contractions.

The experimental trials performed in each condition are shown in Figure 1. In the Short condition, isometric joint torque was obtained at PF0°. In the Control trial, ankle joint angle was set at PF0°. Electrical stimulation was then applied. In the RFE trial, the ankle joint angle was set at PF15°. Electrical stimulation was then applied. Two seconds after initiating stimulation, the ankle joint angle was moved to PF0° with a joint angular velocity of 20°/s. In the Long condition, isometric joint torque was obtained at DF15°. In the Control trial, ankle joint angle was set at DF15°. Electrical stimulation was then applied. In the RFE trial, ankle joint angle was set at PF0°. Electrical stimulation was then applied. Two seconds after initiating stimulation, ankle joint angle was moved to DF15° with a joint angular velocity of 20°/s. In this experiment, the control trials in Short and Long conditions were conducted before the RFE trials in Short and Long conditions. The sequence of the conditions (i.e., Short or Long) was randomized. The interval between trials was at least 2 min to avoid the influence of muscle fatigue on the next trial. During these procedures, the subjects were instructed to keep relaxed state to avoid any redundant voluntary contractions.

### Joint torque and ultrasonographic measurements

Joint torque and joint angle were recorded at a sampling frequency of 4,000 Hz (Power lab 16/30; ADInstruments, Bella Vista, Australia). Isometric joint torques recorded 4.9 s after initiating electrical stimulation (see Figure 1) were used in the statistical analyses. Ultrasonographic measurement (SSD-3500; Aloka, Tokyo, Japan) was performed at the same time as joint torque measurement. Fascicle length and pennation angle of the gastrocnemius were measured using a linear array probe (UST-5710; Aloka, Tokyo, Japan) at a sampling frequency of 30 Hz. Fascicle length was defined as the straight distance between the intersection of the superficial aponeurosis and fascicle and the intersection of the deep aponeurosis and fascicle. Pennation angle was defined as the internal angle formed by the fascicle and deep aponeurosis. The acquired images were analyzed using Image J 1.47v software (National Institutes of Health, Bethesda, MD, US). Fascicle lengths and pennation angles recorded 4.9 s after initiating electrical stimulation were used in the statistical analyses.

### Statistics

To confirm whether residual force enhancement occurred, isometric joint torque values obtained between the Control and RFE trials were compared using paired *t*-tests. In addition, the relative values of joint torque (i.e., joint torque recorded in the RFE trial relative to that recorded in the Control trial) were calculated in both the Short and Long conditions as a magnitude of residual force enhancement. This value was compared between the Short and Long conditions using paired *t*-tests to examine the influence of operating range (i.e., muscle length) on the magnitude of residual force enhancement. Similarly, fascicle lengths and pennation angles obtained during the Control and RFE trials were compared by paired *t*-tests to confirm whether muscle architectural properties affected the differences in joint torque. Statistical analyses were performed using SPSS version 20 software (IBM, Tokyo, Japan) with the level of statistical significance set at *p* < 0.05. All raw data are provided in the Supplementary material.

## Results

### Isometric joint torque

**Figure 2:**
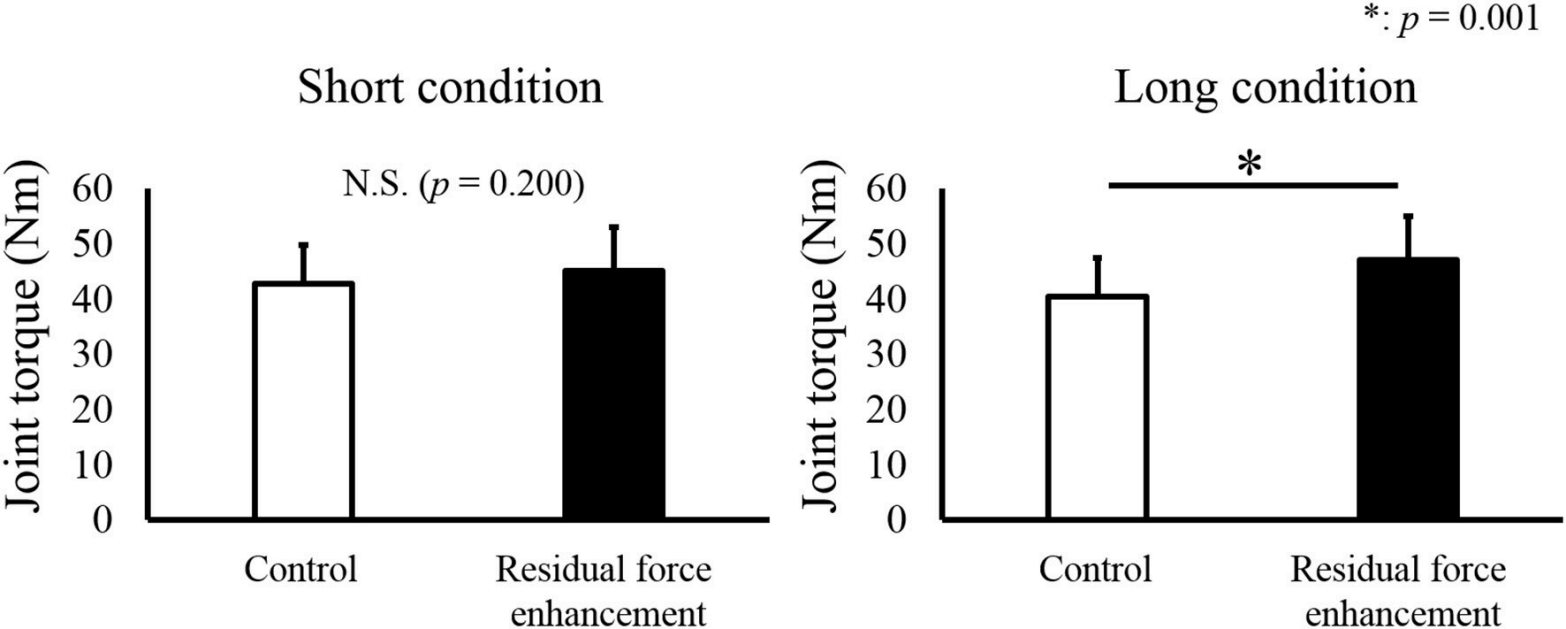
**Joint torque values observed in the Control and Residual force enhancement trials**. Joint torque values observed in the Control trial (pure isometric contraction) are shown as a white bar, and those observed in the Residual force enhancement trial (isometric contraction after eccentric contraction) are shown as a black bar. The left panel shows the joint torque value obtained at plantar flexion (PF) 0° and the right panel shows the joint torque value obtained at dorsiflexion (DF) 15°. Values are presented as means ± standard deviation. ^*^ Indicates a significant difference (*p* < 0.05) between the Control and Residual force enhancement trials.

**Figure 3:**
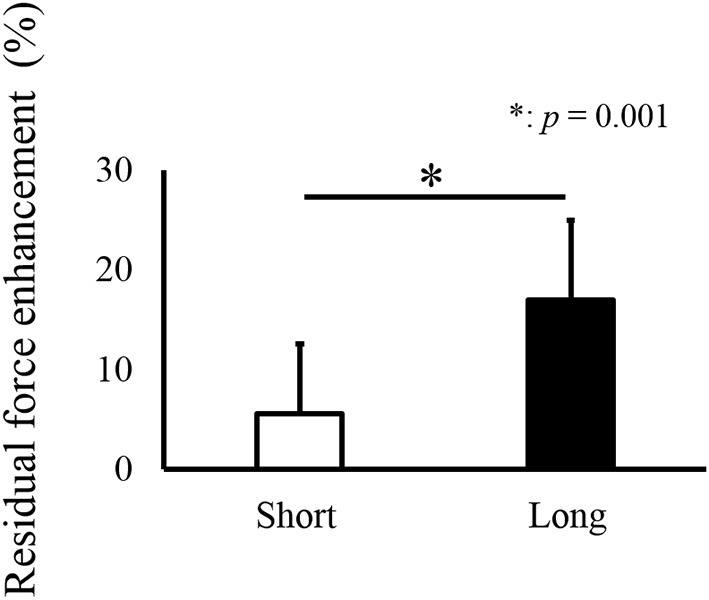
Relative values of joint torque (Residual force enhancement trial relative to Control trial), indicating the magnitude of residual force enhancement ^*^ indicates a significant difference (***p*** < 0.05) in residual force enhancement between the Short and Long conditions.

For isometric joint torque, a paired *t*-test revealed that isometric joint torque was not different between the Control and RFE trials in the Short condition (*p* = 0.200). On the other hand, significant difference between the Control and RFE trials was observed in the Long condition (*p* = 0.001, Figure 2). In addition, relative value of isometric joint torque between the Control and RFE trials, which indicated the magnitude of residual force enhancement, was significantly larger in the Long condition than in the Short condition (*p* = 0.001, Figure 3).

### Fascicle length and pennation angle

**Figure 4:**
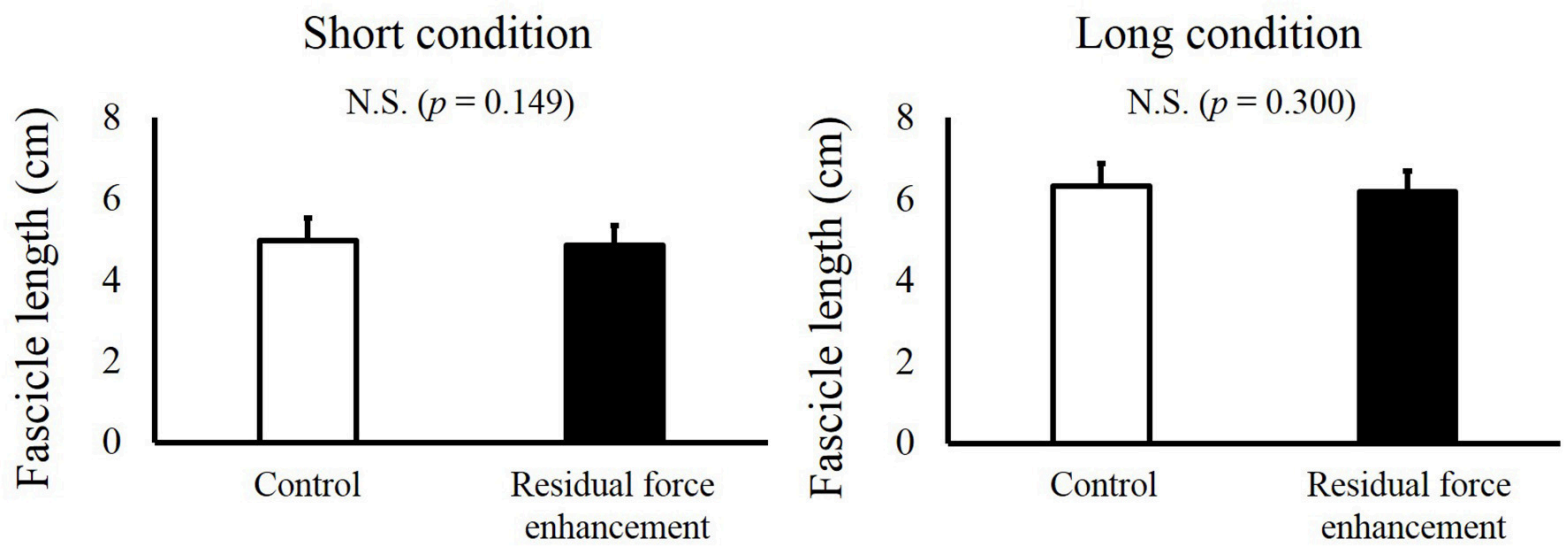
**Fascicle lengths in the Control and Residual force enhancement trials in the Short and Long conditions**. Fascicle lengths observed in the Control trial (pure isometric contraction) are shown as white bars, and those observed in the Residual force enhancement trial (isometric contraction after eccentric contraction) are shown as black bars. The left panel shows the fascicle length obtained at plantar flexion (PF) 0° and the right panel shows the fascicle length obtained at dorsiflexion (DF) 15°. Values are means ± standard deviation.

**Figure 5:**
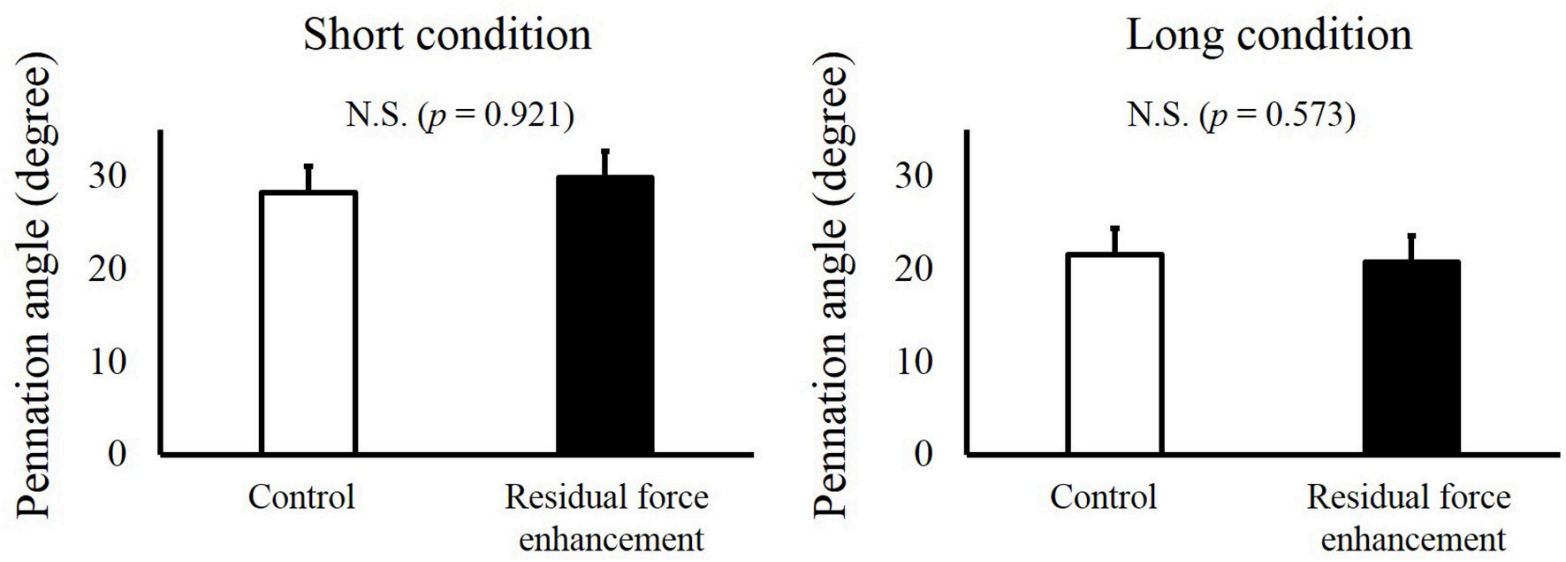
**Pennation angles in the Control and Residual force enhancement trials in the Short and Long conditions**. Pennation angles observed in the Control trial (pure isometric contraction) are shown as white bars, and those observed in the Residual force enhancement trial (isometric contraction after eccentric contraction) are shown as black bars. The left panel shows the pennation angle obtained at plantar flexion (PF) 0° and the right panel shows the pennation angle obtained at dorsiflexion (DF) 15°. Values are means ± standard deviation.

For fascicle length, a paired *t*-test revealed no significant difference between the Control and RFE trials in the Short condition (*p* = 0.149) and Long condition (*p* = 0.300, Figure 4). For pennation angle, paired *t*-test revealed no significant difference between the Control and RFE trials in the Short condition (*p* = 0.921) and Long condition (*p* = 0.573, Figure 5).

## Discussion

In this study, we examined whether residual force enhancement occurs in human plantar flexors, and assessed its dependency on joint angle. We found that in the Long condition, isometric joint torque value obtained in the RFE trial was significantly larger than that obtained in the Control trial. On the other hand, no significant differences were observed in fascicle length and pennation angle between the RFE and Control trials. Therefore, we concluded that the isometric joint torque differences observed were not caused by architectural properties, but force-generating capability of muscle fibers, i.e., residual force enhancement. However, this effect was limited because significant isometric joint torque difference was not observed between the Control and RFE in the Short condition. Therefore, the influence of residual force enhancement in human plantar flexors appears to be dependent on joint angle.

### Joint angle dependence of residual force enhancement in human plantar flexors

Although many studies have confirmed substantial residual force enhancement in isolated muscle preparations (Edman et al., 1978, 1982; Joumaa et al., 2008; Leonard et al., 2010; Rassier and Pavlov, 2012), it remains unclear whether this phenomenon can be applied to human movement in the physiological range of motion. This is because the magnitude of residual force enhancement is strongly dependent on the operating range of the muscle, i.e., residual force enhancement is small in the ascending limb and large in the descending limb (Morgan et al., 2000). Furthermore, the operating region of living muscles is muscle-specific (Burkholder and Lieber, 2001). Many studies have discussed the above points using isolated muscle preparation adopted the descending limb to emphasize the influence of residual force enhancement. On the other hand, human plantar flexors are known to operate only in the ascending limb (Kawakami et al., 1998; Maganaris, 2001). Therefore, the magnitude of residual force enhancement should be small or negligible in human plantar flexors. In fact, although this study confirmed substantial residual force enhancement in the Long condition, no significant residual force enhancement was observed in the Short condition. This result is partly in line with previous study adopted knee extensors (Power et al., 2013). This previous study (Power et al., 2013) compared the magnitude of residual force enhancement in the short and long muscle length conditions, and reported that residual force enhancement was larger in the long muscle length condition than in the short muscle length. However, they also observed significant residual force enhancement even in the short muscle length condition, which is slightly different from our findings. This difference was likely caused by the differences in the operating ranges of the knee extensors and plantar flexors. Specifically, knee extensors work at a relatively long muscle length, which includes not only the ascending limb but also the plateau and descending limb (Herzog et al., 1991). On the other hand, plantar flexors work at a relatively short muscle length, which includes only the ascending limb (Kawakami et al., 1998; Maganaris, 2001). Considering the fact that residual force enhancement is prominent in the descending limb and small in the ascending limb (Morgan et al., 2000), the definition of “short” condition would be different between the previous study (knee extensors, Power et al., 2013) and this study (plantar flexors) because the former would include descending limb while the latter includes the ascending limb only. This explains the lack of residual force enhancement in the Short condition in the current study. In addition, Shim and Garner (2012) reported that residual force enhancement occurred only in long muscle length condition in knee extensors and flexors, which are similar to our results in the plantar flexors. Thus, the result of the current study that in the case of human plantar flexors, the influence of residual force enhancement may be limited to the dorsiflexed region (i.e., long muscle length region) would be valid.

### Influence of muscle architecture

It is possible that the architectural properties of the muscles affected the observed differences in torque in this study. In the case of isolated muscle preparations such as myofibrils and/or single muscle fibers, changes in the observed force simply represent changes in the force-generating capability of the muscle. However, this is not true in the case of changes observed in joint torque. Because some muscles are arranged obliquely with respect to the tendon, a characteristic expressed by the pennation angle (Kawakami et al., 1998), joint torque can be modified when the pennation angle changes. In addition, because muscles connect to bones via tendons and tendon are compliant to some extent (Fukunaga et al., 2002; Fukashiro et al., 2006), joint torque at a given joint angle can be modified by tendon elongation as well. Specifically, even at a given joint angle (i.e., at a given muscle-tendon complex length), the fascicle length (muscle fiber length) is not necessarily consistent (Fukashiro et al., 1995; Ichinose et al., 1997). As a result, the force-generating capability of muscle fibers can be modified according to the force-length relationship (Edman, 1966; Gordon et al., 1966). Considering these points, fascicle length and pennation angle were taken into account in this study. As a result, fascicle length and pennation angle were not different between the Control and RFE trials. These results indicate that architectural properties did not affect the isometric joint torque difference observed in the current study. Of course, because this study adopted relatively-low intensity contractions (25% of the maximal voluntary contraction), the results could be different if higher intensity contractions are used. However, previous studies using 100% of the maximal voluntary contraction observed similar results: no difference in fascicle length and pennation angle (Seiberl et al., 2010; Tilp et al., 2011; Power et al., 2012). Therefore, we believe that architectural properties of a muscle do not affect residual force enhancement.

### Residual force enhancement in humans

Although no significant differences were observed in the Short condition (only 5 subjects out of 11 showed residual force enhancement), significant difference was observed in the Long condition (all subjects showed residual force enhancement). The magnitude of residual force enhancement in the Long condition was 117.0% ± 12.9% (the smallest value was 103.3% and the largest value was 142.5%). These values are in agreement with those of previous studies that employed the same muscles and joint angles (Pinniger and Cresswell, 2007), and other muscles such as human adductor pollicis (Lee and Herzog, 2002; Fortuna et al., 2016), human tibialis anterior (Power et al., 2012). Therefore, our results should be valid, and we conclude that substantial residual force enhancement occurs in human plantar flexors, at least at DF15°. In addition, although no residual force enhancement was observed at PF0° (Short condition), it is possible that residual force enhancement occurs even at PF0° in the different active stretch conditions. In concrete terms, because the magnitude of residual force enhancement is known to be large when the magnitude of active stretch is large (Bullimore et al., 2007), substantial residual force enhancement may occur even at PF0° when active stretch is commenced from a more plantar flexed-joint angle than we adopted (i.e., PF15°). This point should be confirmed in future studies in order to clarify the application of residual force enhancement in human movements.

### Other factors related to residual force enhancement and limitations

Regarding the activation of muscles, this study adopted artificial electrical activation instead of voluntary activation. We believe that our results obtained by the artificial electrical activation can be applied to the voluntary activation as well, which is a physiological condition. This is because residual force enhancement is considered to be derived from titin elasticity and induced by active stretch of muscle fibers and Ca^2+^ release from the sarcoplasmic reticulum (Joumaa et al., 2008), and active stretch and Ca^2+^ release are common events in the both artificial electrical and voluntary activation conditions. In addition, movement modality should be mentioned. Some previous studies adopted multi-joint movements instead of single joint movements for examining whether residual force enhancement is observed in human movements (Seiberl et al., 2013; Paternoster et al., 2016) which seems to be more closely-related to human movements. In these situations, not only agonists but also antagonists should be considered. Specifically, enhanced force generating capability of agonists induced by residual force enhancement does not necessarily contribute to enhancement of performance due to the interaction of synergist and/or antagonists. In addition, fascicle behavior, which is a key for residual force enhancement, is more complicated in multi joint movements. Thus, these points also should be examined in the future.

## Conclusions

In this study, we examined whether residual force enhancement occurs in human plantar flexors and assessed the joint angle dependence of this phenomenon in human plantar flexors. We observed significant residual force enhancement in the Long condition, although no residual force enhancement was observed in the Short condition. These results suggest that residual force enhancement occurs in human plantar flexors, but that its influence in only apparent in the dorsiflexed region. This is due to the limited operating range of the plantar flexors (ascending limb only), and the muscle length dependence of residual force enhancement being smaller in the ascending limb and larger in the descending limb.

## Author contributions

AF and JM performed the experiments, and analyzed the data. AF, JM, and TI wrote the paper.

## Funding

This study was partly supported by the Grant-in-Aid for Challenging Exploratory Research (16K13009) and Postdoctoral Fellowship for Research Abroad (183).

### Conflict of interest statement

The authors declare that the research was conducted in the absence of any commercial or financial relationships that could be construed as a potential conflict of interest.
